# T-Cell Populations and Cytokine Expression Are Impaired in Thymus and Spleen of Protein Malnourished BALB/c Mice Infected with *Leishmania infantum*

**DOI:** 10.1371/journal.pone.0114584

**Published:** 2014-12-23

**Authors:** Sergio Cuervo-Escobar, Monica Losada-Barragán, Adriana Umaña-Pérez, Renato Porrozzi, Leonardo Saboia-Vahia, Luisa H. M. Miranda, Fernanda N. Morgado, Rodrigo C. Menezes, Myriam Sánchez-Gómez, Patricia Cuervo

**Affiliations:** 1 Laboratorio de Hormonas, Departamento de Química, Universidad Nacional de Colombia, Bogotá, Colombia; 2 Laboratorio de Pesquisa em Leishmaniose, Instituto Oswaldo Cruz, Fiocruz, Rio de Janeiro, Rio de Janeiro, Brasil; 3 Instituto de Pesquisa Clínica Evandro Chagas, IPEC, Fiocruz, Rio de Janeiro, Rio de Janeiro, Brasil; INRS - Institut Armand Frappier, Canada

## Abstract

Visceral leishmaniasis (VL) is a parasitic infectious disease that causes significant morbidity and mortality in the tropical and subtropical regions of the world. Although infections with visceralizing *Leishmania* may be asymptomatic, factors such as undernutrition increase the likelihood of progressing to clinical disease. Protein malnutrition, the most deleterious cause of malnutrition in developing countries, has been considered as a primary risk factor for the development of clinical VL. However, data regarding the immunological basis of this association are scarce. With the aim to analyze the effects of protein malnutrition on *Leishmania infantum* infection, we used BALB/c mice subjected to control or low protein isocaloric diets. Each animal group was divided into two subgroups and one was infected with *L. infantum* resulting in four study groups: animals fed 14% protein diet (CP), animals fed 4% protein diet (LP), animals fed 14% protein diet and infected (CPi), and animals fed 4% protein diet and infected (LPi).The susceptibility to *L. infantum* infection and immune responses were assessed in terms of body and lymphoid organ weight, parasite load, lymphocyte subpopulations, and cytokine expression. LPi mice had a significant reduction of body and lymphoid organ weight and exhibited a severe decrease of lymphoid follicles in the spleen. Moreover, LPi animals showed a significant decrease in CD4^+^CD8^+^ T cells in the thymus, whereas there was an increase of CD4^+^ and CD8^+^ T cells percentages in the spleen. Notably, the cytokine mRNA levels in the thymus and spleen of protein malnourished-infected animals were altered compared to the CP mice. Protein malnutrition results in a drastic dysregulation of T cells and cytokine expression in the thymus and spleen of *L. infantum*-infected BALB/c mice, which may lead to defective regulation of the thymocyte population and an impaired splenic immune response, accelerating the events of a normal course of infection.

## Introduction

Visceral leishmaniasis (VL) is a parasitic disease that causes significant morbidity and mortality in tropical and subtropical regions worldwide. The estimated global incidence of VL is approximately 400,000 new cases and 60,000 deaths per year [1]. The main species responsible for VL are *Leishmania donovani* in the Old World and *L. infantum* in Old and New Worlds. Bangladesh, India, Nepal, Sudan, Ethiopia and Brazil are the most affected countries, accounting for ∼90% of VL cases [2]. In Brazil, 3,000–5,000 VL cases occur annually, the majority of which involve children or immunocompromised adults, particularly in the poor agrarian regions of the Northeast [3]. However, VL increasingly affects areas peripheral to urban centers [4]–[8].

The parasite infects cells of the reticulo-endothelial system and primarily affects the spleen, liver, bone marrow and lymph nodes, and results are fatal if left untreated [2]. Although infections with visceralizing parasites may be asymptomatic, factors such as malnutrition increase the likelihood of progressing to clinical disease [9]. malnutrition is responsible for 2.2 million annual deaths worldwide of children under the age of five [10]. Deficiency in protein consumption is likely the most deleterious cause of malnutrition in developing countries where it is frequently related to socioeconomic, political, or environmental factors. More than 300 million children suffer from protein deficiency, and the mortality rate is as high as 40% [11]. Child malnutrition is the main contributor to mortality under the age of five because of the greater susceptibility to infections [12].

Malnutrition has been increasingly implicated in the development of VL clinical manifestations [13]–[15] and has been recognized as an important epidemiological risk factor for the disease [16]. Early studies on malnutrition-VL association showed that children that developed clinical VL presented a precondition of moderate-to-severe malnutrition and that children with such level of malnutrition showed 8.7 times more risk to develop clinical disease than children who are not severely malnourished [9], [17]. Additionally, reduced birth weight was associated to a higher risk of developing VL while increased breastfeeding time was associated with asymptomatic infection [15]. During experimental infections, it was demonstrated that malnourished mice infected with *L. donovani* present a defect in their innate immune responses and early visceralization through functional failure of the lymph node barrier [18]–[20]. In addition, decreased levels of IL-10 and TNF-α and high prostaglandin/leukotriene production have been reported in that model [18], [21]. A decrease in IFN-γ production was also observed in malnourished mice infected with *L. chagasi* [22].

In experimental malnutrition, one of the most remarkable tissue alterations is the thymic atrophy characterized by a reduced cell proliferation and an increased death of double positive T cells [23]–[25]. Such alterations directly impact secondary lymphoid organs involved in the immune response to infections. Notably, the thymus has been barely studied during malnutrition-infection association, and information is limited to *Trypanosoma cruzi* infections [26], [27]. Although several epidemiological and experimental studies have reported an association between malnutrition and an increased risk of developing VL [13]–[22], [28]–[30], data regarding the role of lymphoid organs during this association remain scarce.

In this study, we used a murine model to address the impact of protein malnutrition on *L. infantum* infection. We evaluated the effect of two isocaloric diets supplying distinct levels of protein on the infection of BALB/c mice. Our findings demonstrate the deleterious impact of protein malnutrition on *L. infantum* infection and subsequent changes in lymphocyte subsets, cellularity in lymphoid organs, disruption of spleen micro-architecture and cytokine mRNA expression levels in the thymus and spleen of the animals.

## Materials and Methods

### Ethics statement

The *L. infantum* strain MCAN/BR/2000/CNV-FEROZ used in this study was provided by the Collection of *Leishmania* of the Instituto Oswaldo Cruz, (Coleção de *Leishmania* do Instituto Oswaldo Cruz, CLIOC) (http://clioc.fiocruz.br/). CLIOC is registered in the World Federation for Culture Collections (WFCC-WDCM 731) and is recognized as a Depository Authority by the Brazilian Ministry of the Environment (D.O.U. 05.04.2005). This study was carried out following the recommendations in the Guide for the Care and Use of Laboratory Animals of the National Institutes of Health - Eighth Edition. All animal procedures were approved by the Instituto Oswaldo Cruz and Universidad Nacional de Colombia Animal Care and Use Committees (LW-27/14).

### Parasite culture

Parasites were cultivated at 25°C in Schneider’s medium containing 10% fetal bovine serum (FBS) and were collected at the stationary phase by centrifugation at 1800 *g* for 5 min. The parasites were then washed twice in PBS, pH 7.2.

### Mice, feeding protocol and experimental infection

BALB/c mice (n = 48) were weaned at postnatal day 21 and provided a diet containing 14% protein (MP Biomedicals, Inc., USA, Catalog No. 960258) for a 1 week of pre-adaptation period. The mice were housed three per cage and maintained in a 12/12 h light/dark cycle at a constant temperature. After that 1 week of acclimation, the mice were randomly divided into two groups: 24 animals were fed a 14% protein diet (13,79 g crude protein per 100 g food pellets; control protein, CP) and 24 animals were fed a 4% protein diet (4,59 g crude protein per 100 g food pellets; low protein, LP) (MP Biomedicals, Inc., USA, Catalog No 960254). The diets were isocaloric, with each providing 3.7 kcal/g. The caloric protein deficiency in the 4% protein diet was replaced by additional carbohydrate calories. The animals had free access to water and food. Food rations per cage were daily weighed and feed consumption was calculated [31]. Diets were chosen based on previous reports that 4% protein diet induces protein deficiency but not energy deficiency whereas 14% protein diet has been reported to provide standard protein requirements for supporting normal growth in mice [32]. After 7 days of diet, each animal group was divided into two subgroups; one was infected intravenously (tail vein) with 1×10^7^ parasites, whereas the other group received saline solution, resulting in the following four study groups: animals fed 14% protein diet (CP), animals fed 4% protein diet (LP), animals fed 14% protein diet and infected (CPi), and animals fed 4% protein diet and infected (LPi). The diets were maintained after infection. Body weight was measured daily during the experimental course (21 days). For ethical considerations, experiment was not extended over 21 days as the body weight loss was already at the allowed limit permitted by the Animal Care and Use Guidelines. The infection course lasted 14 days. The animals were euthanized after 14 days post-infection. Euthanasia was conducted according to the protocol approved by license LW-27/14. Briefly, animals were anaesthetised intraperitoneally with a mix of 10 mg/kg xylazine - 200 mg/kg ketamine. When anaesthetized, the animals were exposed to carbon dioxide gas. Blood was collected by cardiac puncture, and the sera were separated and stored at −20°C. The spleen, thymus, and liver were quickly removed, weighed, and subsequently processed for parasite culture, cell isolation or nucleic acid extraction. Splenocytes or thymocytes suspension were obtained by injection of PBS and gentle washes of the respective organs with sterile needle syringes. Intact cells were pelleted by centrifugation and supernatants were recovered for soluble cytokine analysis, these supernatants are considered as enriched interstitial fluids. Relative and absolute cell number in thymus and spleen were estimated by haemocytometer counting. The relative weight of the tissues at the sacrifice day was calculated as the tissue weight (g)/body weight (g)×100. The bone marrow was also extracted from the femurs of both legs.

### Evaluation of infection positivity and determination of parasite load

Samples from spleen, thymus, and liver from each infected animal were washed twice in sterile PBS and incubated at 25°C in biphasic culture medium (Novy-MacNeal-Nicolle (NNN)/Schnneider) supplemented with 10% FBS [33]. In addition, aliquots of bone marrow and blood from each infected animal were also cultured. Positivity was determined by the presence of proliferative promastigote forms in the culture. Positivity of infection was also verified in the liver and spleen by real-time quantitative PCR (qPCR). This assay was also used to determine the parasite load in these tissues. DNA from the liver and spleen samples was extracted using a commercial kit. For accurate sensitivity, kinetoplast DNA (kDNA) was used as target for amplification with the TaqMan system. In vitro culture of *L. infantum* promastigotes was used to construct the standard curves and assess both the sensitivity and efficiency of the assay. kDNA copy numbers were normalized to the *Ubiquitin C* (*UBC*) gene from mice and adjusted to the number of cells detected in the tissue of each animal. Primer sequences are shown in the S1 Table.

### Histopathological and immunohistochemical analysis

Spleen, liver and bone marrow fragments were fixed in 10% buffered formalin, embedded in paraffin and sliced in 5-µm thick sections mounted on microscope slides. The sections were stained with haematoxylin and eosin (HE) and examined by light microscopy (Nikon Eclipse E400– Tokyo, Japan). The degree of white pulp structural organization of the spleen was analyzed as described by Santana et al (2008) [34]. Briefly, the splenic white pulp organization was classified as 1- well organized – with distinct peri-arteriolar lymphocyte sheath, germinal centre, mantle zone and marginal zone, and ≥3 follicles/mm^2^; 2- slightly disorganized - with either hyperplastic or hipoplastic changes leading to a loss in definition of any of the regions of the white pulp; and ≥3 follicles/mm^2^; 3- moderately disorganized – when the white pulp was evident, but its regions were poorly individualized or indistinct; and >1 to <3 follicles/mm^2^; and 4- highly disorganized – when the follicular structure was barely distinct from the red pulp and T-cell areas, ≤1 follicle/mm^2^. The follicles were quantified in at least 5 microscopic fields under 100x magnification using a grid-scale with 20×20 subdivisions in an area of 10 mm^2^. The median of follicle number was compared among groups. Additional microscopic sections were also placed on silanated slides for immunohistochemistry against *L. infantum*. These slides were treated as described previously [35].

### Lymphocyte subpopulation analysis

Splenocytes and thymocytes were obtained by PBS washes of the respective organs. The cells (1×10^6^) were stained with anti-mouse CD3 APC-conjugated, CD4 FITC-conjugated, or CD8a PerCP-conjugated antibodies plus isotype IgG control antibodies. Acquisition (10,000 events) was performed in a FACScan flow cytometer. Off-line analysis was performed with Summit Software, version 4.0.

### Gene expression analysis

Total RNA was extracted from cells by using Trizol Reagent according to the manufacturer’s instruction. The quantity and integrity of RNA were determined by spectrophotometer (Nanodrop ND-1000, NanoDrop Technologies); RNA samples with OD 260/280 of approximately 2.0 were used for qPCR. DNA was synthesized from 1 µg of total RNA using SuperScript III reverse transcription system and oligo-dT primers in total reaction volumes of 20 µL. Real time PCR was performed in duplicate using the 7500 Applied Biosystems equipment and SYBR Green PCR Master Mix, according to the manufacturer’s protocol. Oligonucleotide sequences of the target genes *TNF-α, IFN-γ, TGF-β, IL-10, IL-12, IL-4*, in addition to reference genes *gapdh, atp-5 and cyc-1* (S1 Table) were designed using the Vector NTI software from available public sequences. PCR conditions for all primers were optimized and specificities were verified by melting curve analyses and agarose gel electrophoresis. The cDNAs were amplified by PCR for 40 cycles consisting of 10 s of denaturation at 95°C, 15 s of annealing at 56°C, and 10 s of extension at 72°C. Gene expression was quantified by means of the comparative Ct method (ΔΔCt). To assess linearity of the assay over an extended range, a standard curve for each gene was generated using serial dilutions of pooled cDNAs from all samples. The efficiency of amplification for each gene was obtained from the manufacturer’s software in the exponential phase of the amplification curve. Data are shown as normalized ratios between target gene expression and geometric media of the three reference genes [36]. All data was presented as mean ± SEM. Experiments were performed following the MIQE guidelines [37].

### Detection of cytokines by Luminex

The cytokines secreted by thymocytes and splenocytes was quantified in the interstitial fluid of these organs using the Luminex technology [38], [39]. The detection was made for: IFN-γ, IL-10 and IL-12 (p40/p70). The fluorescence levels of each molecule were measured and the data analysis performed using the software supplied by the manufacturer. A series of recombinant cytokines from 51 to 8.000 pg/mL were used to establish standard curves and assay sensitivity.

### Statistical analysis

Statistical analysis was performed using GraphPad Prism 5.0 software. A Two-way analysis of variance (ANOVA) was used, in conjunction with the Bonferroni post-test, to analyze differences among the treatments. The Student *t* test was used to analyze differences between body weight due to diet treatments (CP and LP) before infection, and between parasite load data of CPi and LPi animals. The data are presented as the mean ± SEM. Statistical differences among histopathological data, specifically regarding splenic white pulp organization and follicle number were analyzed with the Fisher’s exact test and Mann Whitney test, respectively.

## Results

### Protein malnutrition decreases body and tissue weight in BALB/c mice infected with *L. infantum*

**Figure 1:**
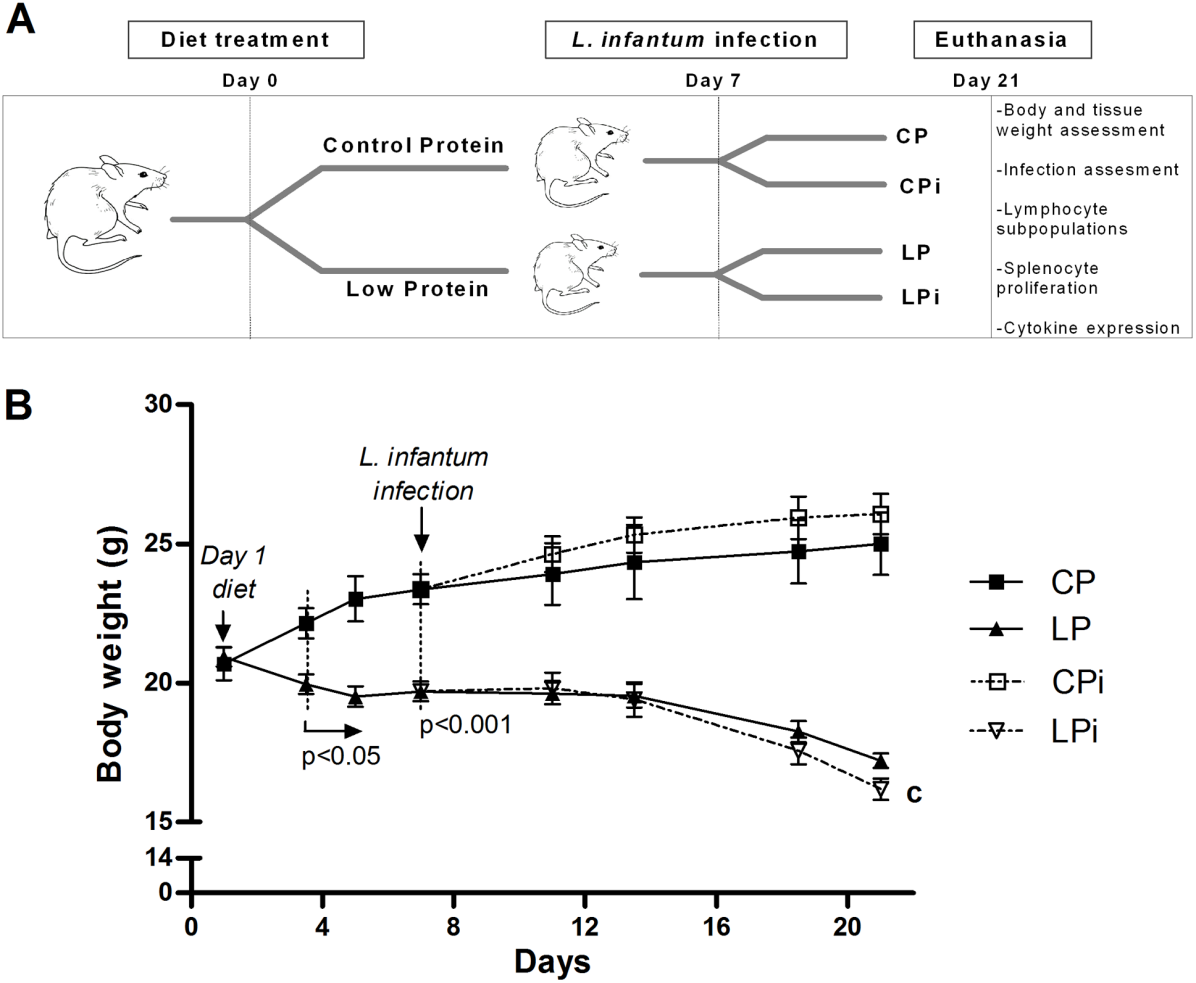
Effect of protein malnutrition on body weight in mice infected with *L. infantum*. (**A**) Schematic representation of the experimental design. BALB/c male mice were fed a 14% (n = 24, CP) or 4% (n = 24, LP) protein diet for 21 days. On day 7 of the experimental period, half the animals were infected with *L. infantum* and the other half received a saline solution injection. CP: animals fed 14% protein diet; LP: animals fed 4% protein diet, CPi: animals fed 14% protein diet and infected; LPi: animals fed 4% protein diet and infected. Two weeks later, the animals were sacrificed and assessed for susceptibility to infection and immunological parameters. (**B**) Body weight was registered every third or four day and expressed as average ± SEM; n = 12 mice in each group. Statistical differences before the day of infection were determined by Student’s *t* test. After infection, a Two-way ANOVA analysis with Bonferroni pos-hoc test was used; **c** = significant interaction between diet and infection in LPi animals at day 21, p<0.05.

**Figure 2:**
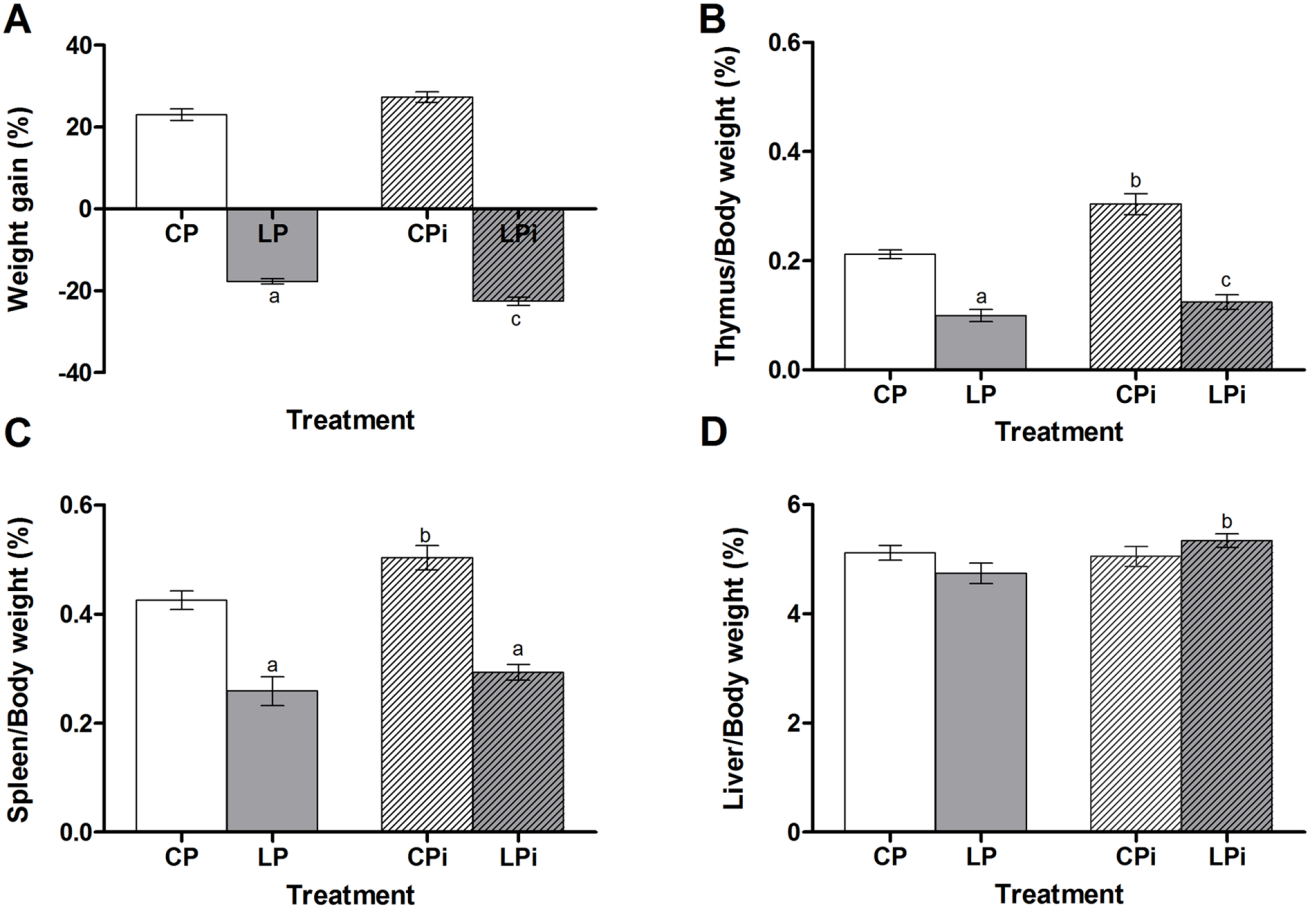
Effect of protein malnutrition on tissue weight in mice infected with *L. infantum*. (**A**) Body weight gain, (**B**) thymus, (**C**) spleen and (**D**) liver weight gain at day 21 expressed as a percentage of tissue/body weight in grams ± SEM (n = 12). Two-way ANOVA analysis with Bonferroni pos-hoc test. Statistical differences due to diet: **a** (p<0.001), infection: **b** (p<0.05) and interaction between diet and infection: **c** (p<0.001). CP: animals fed 14% protein diet; LP: animals fed 4% protein diet, CPi: animals fed 14% protein diet and infected; LPi: animals fed 4% protein diet and infected.

Mice subjected or not to protein malnutrition were randomly divided into two subgroups each, infected or uninfected, and euthanized (Fig. 1A). Mean intakes of the CP and LP diets were comparable: 79.80 (SEM 1.45) g/21d/animal and 76.59 (SEM 2.82) g/21d/animal, respectively. Since the diets were isocaloric, the intakes of metabolizable energy were comparable. Body weight difference between the two dietary groups was significant from day three of the diet (*p*<0.005), increasing through the period of consumption of this. At the day of infection (day seven of diet), LP mice had 16% less body weight (*p*<0.0005) compared to CP animals. Following a previously proposed scale of murine malnutrition, based on weight-for-age (WA) [19], our model showed at this day (seven day) signs of mild malnutrition. This scale, designed by analogy to the classification of human malnutrition [40], defines mild malnutrition as 75–90% WA, whereas 60–75% WA and <60% WA are considered moderate and severe malnutrition, respectively. In contrast, at day 21, LP animals presented a significant 29.8% less body weight compared to CP mice at the same day (*p*<0.0001). A significant interaction between diet and infection variables was observed in the weight of LPi mice at the 21 day of experiment (*p*<0.05) (Figs. 1B and 2A). At this time point, protein restriction caused a moderate malnutrition that rapidly limits with the severe condition (∼36% weight loss). These data of gain or loss of weight are also shown in the Fig. 2A, where the changes in body weight are represented related to the day 1 of diet in each group. Following the trend of body weight loss, after day 21 it would be expected an increased body weight reduction which would be out of the ethical conditions approved for our model, for this reason, the experiment coursed until day 21.

A weight loss was also observed for the relative weight of the lymphoid organs in the LP mice, resulting in a significant weight loss of 47% and 36%, for thymus (*p*<0.001) and spleen *(p*<0.01), respectively, compared to the CP animals at 21 days of dietary protein restriction (Fig. 2B and 2C). Conversely, the weight of thymus and spleen of well-nourished animal is increased due to the *Leishmania* infection (*p*<0.001 and *p*<0.05, respectively). However, the malnourished and infected animal could not increase the weight of these organs (Fig. 2B and C). The weight of the hepatic tissue from the LPi animals was significantly increased as an effect of infection (*p*<0.05) (Fig. 2D). Other organs such as kidney and lungs remained comparables among the groups (data not shown).

### Protein malnutrition induces an increased parasite load in the spleen

**Figure 3:**
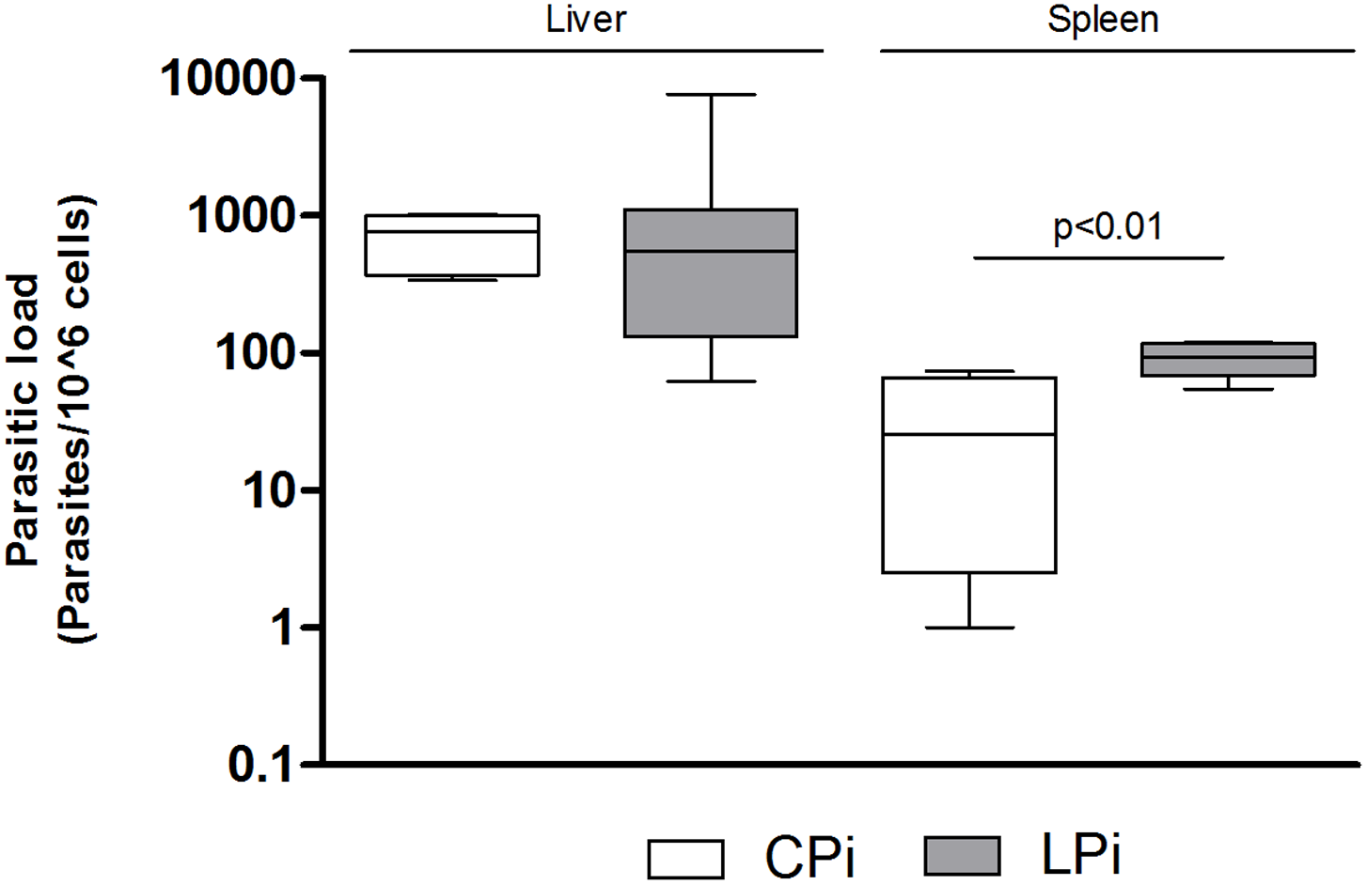
Splenic and liver parasite load in mice infected with *L. infantum*. The parasite load was determined by qPCR in liver and spleen. The number of parasites was calculated in relation to 10^6^ cells. Data represent the mean of 8–10 animals in each group. Statistical differences were analyzed by Student’s *t* test (p<0.01). CPi: animals fed 14% protein diet and infected; LPi: animals fed 4% protein diet and infected.

To analyze the consequences of protein malnutrition on mice infection we examined the presence of the parasite in the liver, spleen, bone marrow and blood of infected animals using one or two distinct methods: *in vitro* culture and qPCR. Samples were obtained from infected mice euthanized 14 days post-infection (dpi) with *L. infantum*. After 14 dpi, it was possible to detect parasites in all the analyzed tissues (Table 1) by one or two methods used. By the less sensitive in vitro culture we observed that 54% of the infected mice (13/24) exhibited liver infection, 67% (16/24) spleen infection, 17% (04/24) bone marrow infection and 4% (01/24) blood infection (Table 1). When liver and spleen samples were analyzed by a more sensitive method, qPCR, we observed that 100% (24/24) were positive in spleen and 83% of the animals (20/24) were positive in the liver (Table 1), indicating that our infection was successful. Further, we wanted to analyze if protein malnutrition could influence the parasite load in spleen and liver of infected animals after 14 dpi. Interestingly, the parasite load obtained by qPCR was similar between the CPi and LPi mice for the liver, but there was a significant increase in the parasite load per 10^6^ cells in the spleens of mice subjected to protein malnutrition (*p* = 0.018, Fig. 3). In fact, according to qPCR data, spleens from LPi animals showed three times the parasite load observed in CPi mice.

**Table 1:** Positivity of infection with *Leishmania infantum* in mice submitted or not to protein malnutrition.

Tissue	Positive detection	
	CPi	LPi
**qPCR**		
Spleen	100% (12/12)	100% (12/12)
Liver	83% (10\12)	83% (10\12)
**Tissue culture**		
Spleen	67% (8\12)	67% (8\12)
Liver	50% (6\12)	58% (7\12)
Bone marrow	17% (2\12)	17% (2\12)
Blood	0% (0\12)	8% (1\12)

CPi: animals fed 14% protein diet and infected; LPi: animals fed 4% protein diet and infected.

### Protein malnutrition induced a significant decrease of lymphoid follicles in the spleens of *L. infantum*-infected mice

**Figure 4:**
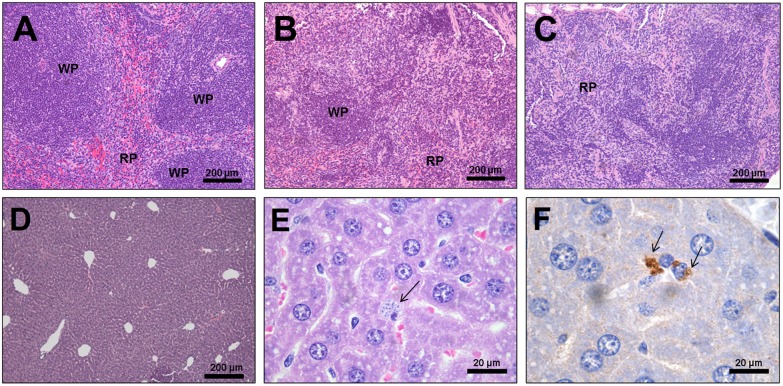
Histopathological alterations in the spleen and liver. Representative images of a spleen with (**A**) organized white pulp, (**B**) moderately disorganized white pulp, and (**C**) highly disorganized white pulp. The decrease or absence of lymphoid follicles is evident in B and C. (**D**) Representative image of liver from CP mice. The presence of amastigotes (arrows) in the CPi liver detected with (**E**) hematoxylin and eosin stain and (**F**) immunohistochemistry. WP: white pulp; RP: red pulp.

Histopathological analysis revealed a significant reduction in the median of splenic lymphoid follicles number in 92% of the LPi mice (11/12), including four animals that showed the absence of lymphoid follicles. The medians of follicle number/mm^2^ were as follows: CP = 3.7 (1.5–4.0); LP = 3.75 (0.5–5.5); CPi = 3.15 (1.0–4.3); LPi = 0.25 (0–2.7) [Mann Whitney test: CP×LP (*p* = 0.77); CP×CPi (*p* = 0.70); LP×LPi (***p*** **= 0.002**); CP×LPi (***p*** **= 0.002**); CPi×LPi (***p*** **= 0.0005**)] (Table 2, Fig. 4). The splenic white pulp of LPi mice showed a significant disorganization characterized by follicular regions poorly individualized or indistinguishable from the red pulp and T-cell areas, and 0 to 2.7 follicles/mm^2^. Splenic disorganization in LPi was significantly different from the other experimental groups [CP×CPi (*p* = 0.60; OR: 3.5); CP×LP (*p* = 0.57; OR: 4.2); LP×LPi (***p*** **= 0.007; OR: 25.0**); CPi×LPi (***p*** **= 0.001; OR: 30.0**); CP×LPi (***p*** **= 0.0002; OR: 105.0**)] (Table 2). Mild hepatic vacuolar degeneration (28%, 4/12) was observed in the LPi animals. In addition, amastigotes were detected by immunohistochemistry in the liver samples of 17% (2/12) of the CPi mice (Fig. 4).

**Table 2:** Organization of the splenic white pulp in mice submitted to protein malnutrition and infected with *Leishmania infantum*.

White pulp	Experimental groups			
	CP	LP	CPi	LPi
Organized to slightly disorganized* (number of animals)	87.5% (7/8)	62.5% (5/8)	66.7% (8/12)	6.3% (1/12)
Moderately to highly disorganized* (number of animals)	12.5% (1/8)	37.5% (3/8)	33.3% (4/12)	92% (11/12)
Follicles/mm^2^ – median (min–max)**	3.7 (1.5–4.0)	3.75 (0.5–5.5)	3.15 (1.0–4.3)	0.25 (0–2.7)

CP = animals fed 14% control protein diet; LP = animals fed 4% protein diet; CPi = animals fed 14% protein diet and infected; LPi = animals fed 4% protein diet and infected control protein and infected.

*Fisher’s exact test: CP×CPi (p = 0.60; OR: 3.5); CP×LP (p = 0.57; OR: 4.2); LP×LPi **(p = 0.007; OR: 25.0)**; CPi×LPi **(p = 0.001; OR: 30.0)**; CP×LPi **(p = 0.0002; OR: 105.0)**.

**Mann-Whitney test: CP×LP p = 0.77; CP×CPi p = 0.70; LP×LPi **p = 0.002**; CP×LPi **p = 0.002**; CPi×LPi **p = 0.0005**.

### Protein malnutrition significantly decreases cellularity and alters the percentage of lymphocyte subpopulations in the thymus and spleen of mice infected with *L. infantum*

Compared to organs such as liver, lungs and kidney that not suffered any change, the weight of thymus and spleen was significantly affected by the diet. This observation prompted us to investigate the effects of protein malnutrition on cellularity in the spleen and thymus of mice subjected to *L. infantum* infection. Although the relative cell number was similar between groups, it was evident that there was an increase in the total cell number of thymus and spleen from CPi mice in response to infection (*p*<0.001) (Table 3). This response was affected in the protein restricted animals; the absolute number of splenocytes and thymocytes from LP mice was significantly lower (*p*<0.001) than the CP group, with a loss of ∼57% or and ∼82%, respectively. This cell loss was worst in the spleens of LPi mice (∼66%), indicating a combined effect between diet and infection (*p*<0.0001) (Table 3).

**Table 3:** Effect of protein malnutrition on *L. infantum* infection in spleen and thymus cellularity of BALB/c mice.

Treatment	Relative cell number (cell/mg of tissue)		Absolute cell number		Percentage of cell loss or gain respect to CP diet	
	Spleen x10^5^	Thymus x10^6^	Spleen x10^7^	Thymus x10^7^	Spleen	Thymus
CP	6.14±0.54	1.13±0.15	6.08±0.63	5.72±0.75	-	-
LP	6.15±0.71	0.91±0.18	2.62±0.33^a^	1.06±0.18^a^	–56.9±5.49	–81.5±3.16
CPi	6.93±0.36	1.56±0.27	8.80±0.73^b^	8.71±0.71^b^	+44.7±12.1	+52.5±12.4
LPi	5.91±0.79	1.10±0.27	2.08±0.30^ac^	1.05±0.23^ac^	–65.8±5.00	–81.7±4.04

CP: animals fed 14% protein diet; LP: animals fed 4% protein diet, CPi: animals fed 14% protein diet and infected; LPi: animals fed 4% protein diet and infected. Letters indicate statistical differences using a Two-way ANOVA analysis with Bonferroni pos-hoc test. Statistical differences due to diet: **a** (p<0.001), infection: **b** (p<0.05) and interaction between diet and infection: **c** (p<0.05).

**Figure 5:**
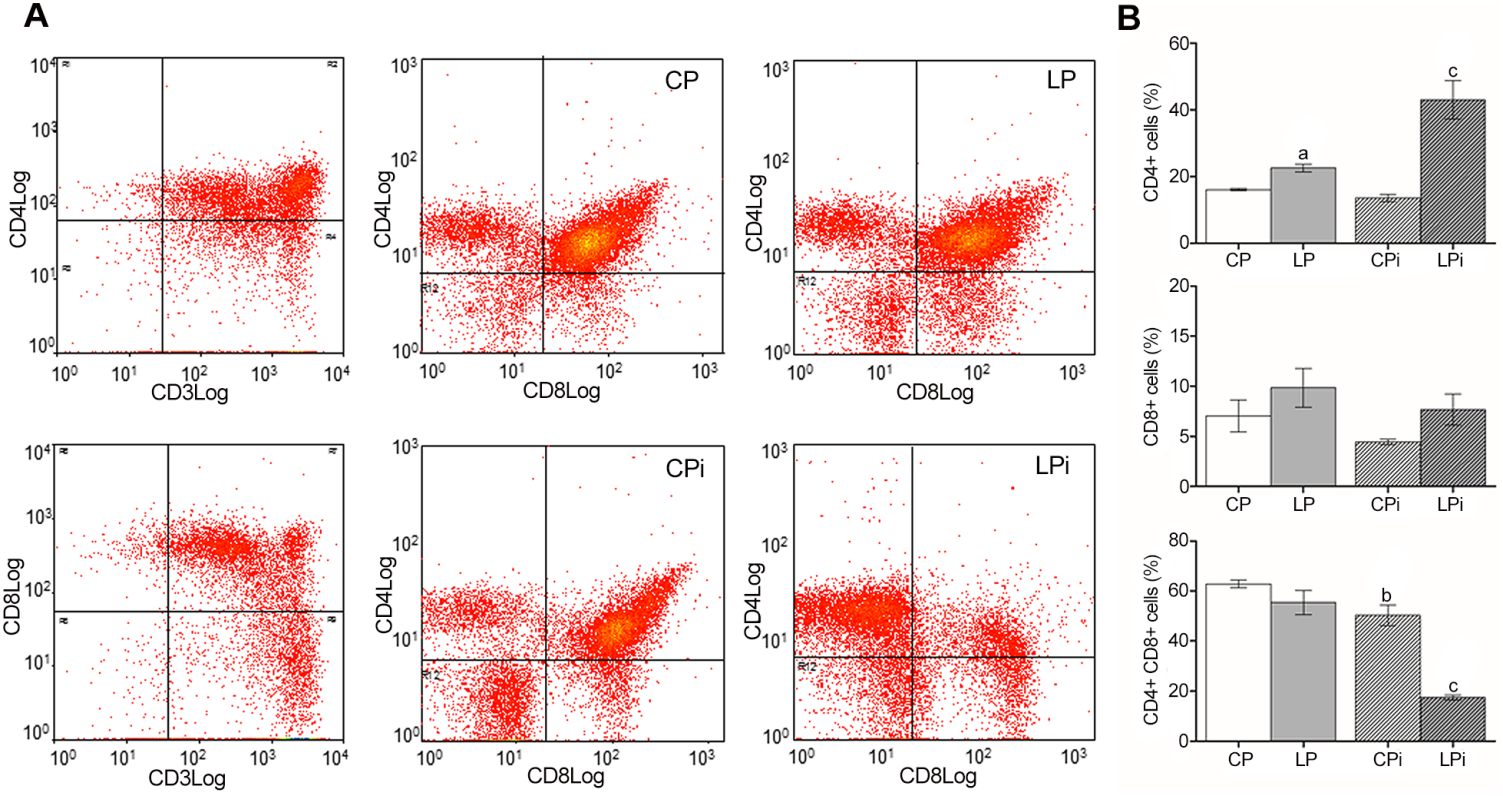
Protein malnutrition dysregulated lymphocyte subpopulations in the thymus of mice infected with *L. infantum*. Lymphocyte subpopulations from the thymus of the experimental groups were measured using FACS analysis as described in the Materials and Methods. (**A**) Representative scatter plots of lymphocyte subpopulations. (**B**) Distribution of lymphocyte subsets expressed as percentage ± SEM. CP: animals fed 14% protein diet; LP: animals fed 4% protein diet, CPi: animals fed 14% protein diet and infected; LPi: animals fed 4% protein diet and infected. Two-way ANOVA analysis with Bonferroni pos-hoc test. Statistical differences due to diet: **a** (p<0.05), infection: **b** (p<0.05) and interaction between diet and infection: **c** (p<0.05).

**Figure 6:**
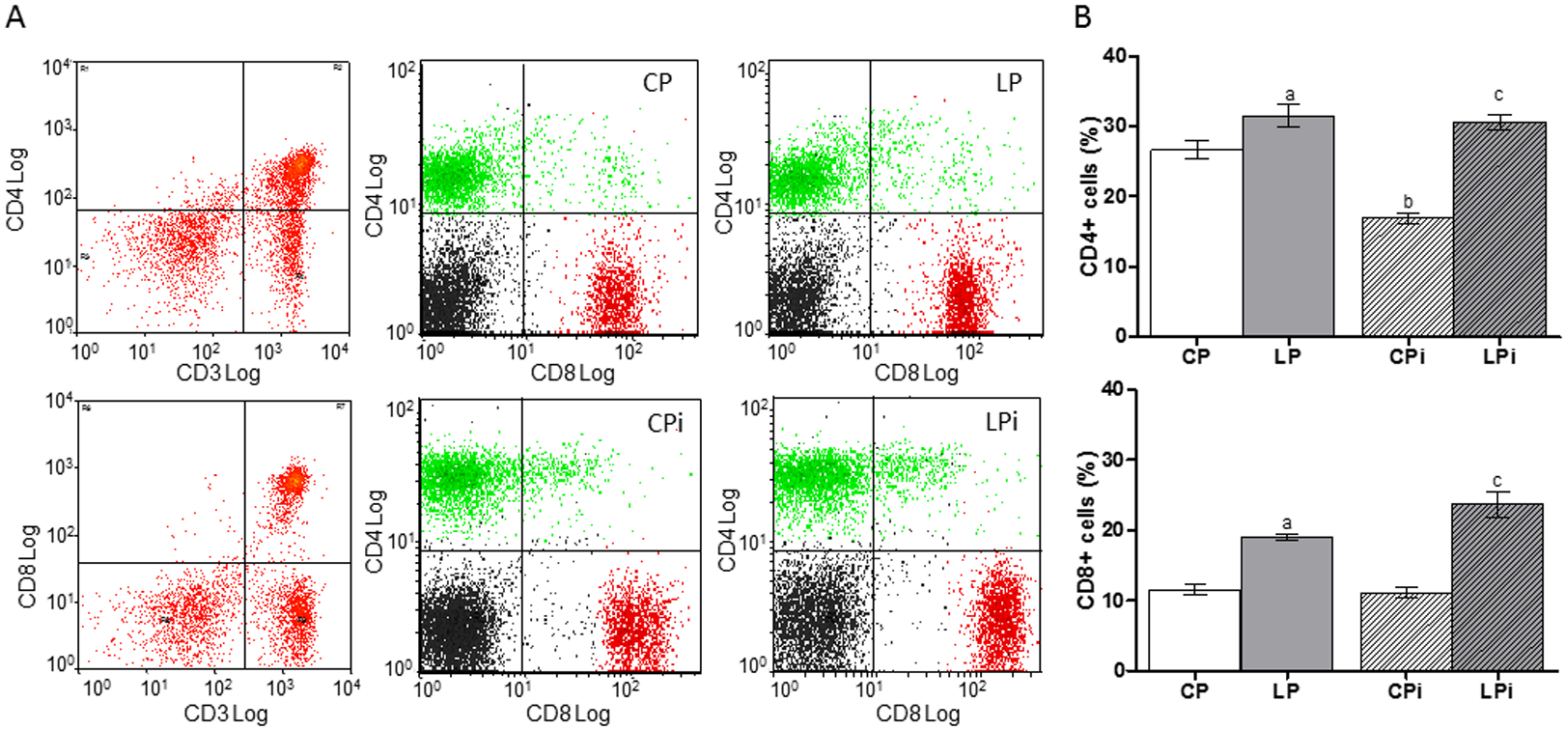
Protein malnutrition dysregulated lymphocyte subpopulations in the spleen of mice infected with *L. infantum*. Lymphocyte subpopulations from the spleen of the experimental groups were measured using FACS analysis as described in the Materials and Methods. (**A**) Representative scatter plots of lymphocyte subpopulations. (**B**) Distribution of lymphocyte subsets expressed as percentage ± SEM. CP: animals fed 14% protein diet; LP: animals fed 4% protein diet, CPi: animals fed 14% protein diet and infected; LPi: animals fed 4% protein diet and infected. Two-way ANOVA analysis with Bonferroni pos-hoc test. Statistical differences due to diet: **a** (p<0.001), infection: **b** (p<0.05) and interaction between diet and infection: **c** (p<0.05).

The severe loss of cellularity in the lymphoid tissues of protein restricted animals led us to analyze whether the distribution of lymphocyte subpopulations was also affected. FACS analysis revealed that the percentage of cells in both organs is affected by protein malnutrition. The percentage of CD4^+^ T cells was significantly increased in the thymus of LP as an effect of the diet (*p*<0.001) and in LPi animals as a combined effect of diet and infection conditions (*p*<0.05) (Fig. 5). Interestingly, the percentage of double positive T cells (CD4^+^CD8^+^ T cells) in the thymus of LPi animals was significantly decreased (*p*<0.001) as a result of interaction between low protein diet and infection with *L. infantum* (Fig. 5). An increase in the percentage of splenic CD4^+^ and CD8^+^ T cells due the interaction between two treatments was observed in LPi mice (*p*<0.001 and *p*<0.05, respectively) (Fig. 6).

### Protein malnutrition dysregulated cytokine expression in the thymocytes and splenocytes of *L. infantum*-infected mice

**Figure 7:**
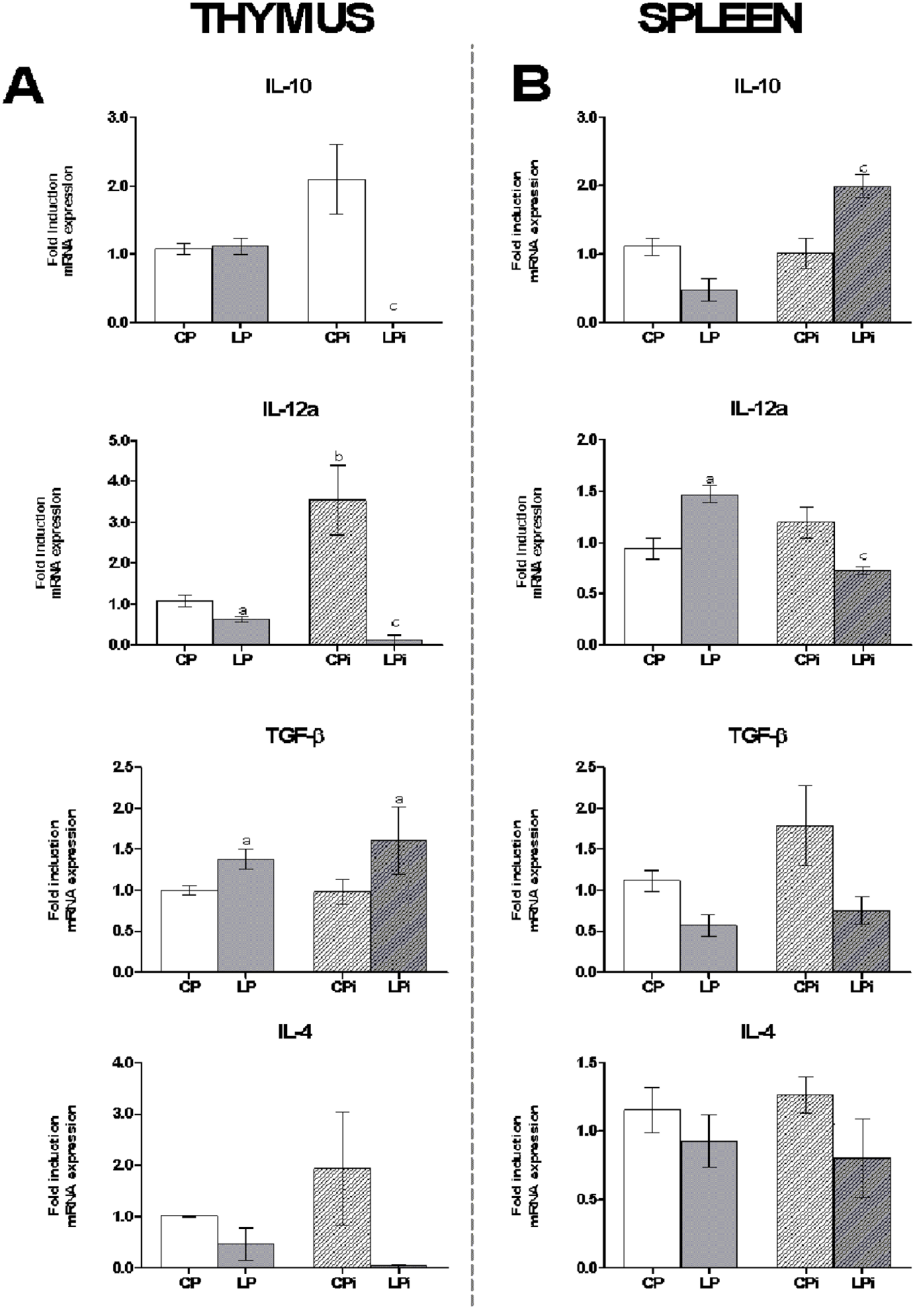
Protein malnutrition dysregulated cytokine expression in thymocytes and splenocytes of mice infected with *L. infantum*. *IL-10, IL-12a, TGF-β, IL-4* and *IFNγ* mRNA expression levels were measured by qPCR in (**A**) thymocytes and (**B**) splenocytes of each experimental group. The values are expressed as normalized ratios between the target gene expression and the geometric median of the *ATP-5, GAPDH* and *CYC-1* genes. The values are expressed in pg/mL ± SEM. CP: animals fed 14% protein diet; LP: animals fed 4% protein diet, CPi: animals fed 14% protein diet and infected; LPi: animals fed 4% protein diet and infected. Two-way ANOVA analysis with Bonferroni pos-hoc test. Statistical differences due to diet: **a** (p<0.001), infection: **b** (p<0.05) and interaction between diet and infection: **c** (p<0.05).

To determine whether protein malnutrition could also affect cytokine mRNA expression in thymocytes and splenocytes, qPCR assays were performed. A significant increase in TGF-β was observed in the thymocytes of animals subjected to protein malnutrition (Fig. 7A). Additionally, IL-10 and IL-12a mRNA levels were significantly reduced in the LPi mice due to the interaction between low protein diet and infection (*p*<0.01) (Fig. 7A). IFN-γ was undetectable under all conditions tested, and the expression data of the other genes showed no significant differences. The mRNA expression levels of TGF-β and IL-10 were downregulated in the splenocytes of the LP mice (Fig. 7B). However, as an effect of interaction, mRNA levels of IL-10 were increased in LPi mice (*p*<0.001) whereas IL-12a levels were decreased (*p*<0.05). In addition, an increased expression of IFN-γ levels in spleen was observed by diet (*p*<0.001) or infection (*p*<0.05), but not by the interaction of these conditions (Fig. 7B).

**Figure 8:**
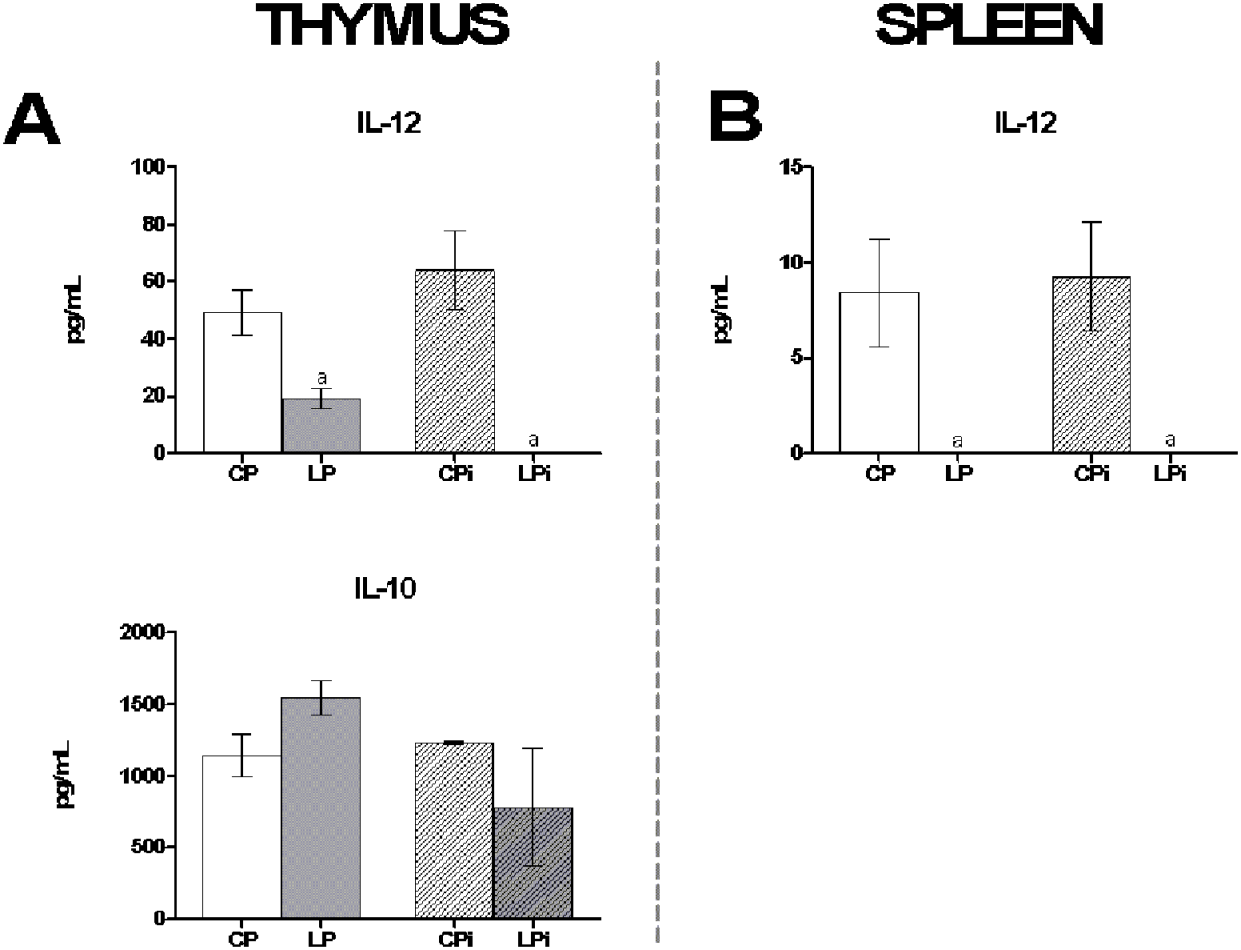
Protein malnutrition dysregulated secreted cytokine levels in the interstitial fluid of thymus and spleen of mice infected with *L. infantum*. *IL-10* and *IL-12* protein levels were measured by Luminex in the interstitial fluid of (**A**) thymus and (**B**) spleen. The values are expressed in pg/mL ± SEM. CP: animals fed 14% protein diet; LP: animals fed 4% protein diet, CPi: animals fed 14% protein diet and infected; LPi: animals fed 4% protein diet and infected. Two-way ANOVA analysis with Bonferroni pos-hoc test. Statistical differences due to diet: **a** (p<0.001).

In addition, the protein levels of IL-10, IL-12 and IFN-γ were quantified in the interstitial fluid obtained from thymus and spleen. IL-12 protein levels were significantly reduced as a consequence of protein restricted diet in the interstitial fluid of the thymus and spleen (*p*<0.001) of LP and LPi mice (Fig. 8A and 8B). In thymus, the protein levels of IL-10 were not significantly distinct among the different treatments (Fig. 8A), whereas in the spleen were undetectable. In addition, IFN-γ was undetectable in the fluids of both organs.

## Discussion

Protein malnutrition is recognized as a critical determinant of impaired immunity and increases the susceptibility to multiple infectious diseases [41]. However, the mechanisms by which protein malnutrition impairs immune function are not completely characterized. In this study, we examined the consequences of protein restriction on *L. infantum* infection in BALB/c mice. We found that protein malnutrition resulted in severe body weight loss affecting primarily the lymphoid organs of *L. infantum*-infected mice. We observed that in response to infection, mice fed on control protein diet (CPi) increased their the total cell number of thymus and spleen. However, protein restricted diet affected this response inducing a significant reduction of the thymic and splenic cell populations in LPi mice, which was accompanied by tissue atrophy. It has been shown that defects on cell proliferation, cell migration and/or increased cell death could account for the reduction of thymic cells due to malnutrition and that such defects have deleterious consequences in the response to infections [24], [27], [42]. In fact, using a murine model of malnutrition, it was observed that the thymic atrophy is largely due to massive thymocyte death combined with decreased thymocyte proliferation primarily due to the loss of immature CD4^+^CD8^+^ double positive T cells [43]. Here, we show data on the conspicuous effect of protein malnutrition on the thymus of *L. infantum*-infected animals. We found that CD4^+^CD8^+^ double positive T cells in the thymus were significantly reduced in protein restricted mice subjected to *L. infantum* infection. Several reasons may account for this phenomenon. First, a deficit of precursors because of defective hematopoiesis could be responsible for the decrease in the percentage of this population. Accordingly, it has been shown that protein-restricted mice have compromised hematopoiesis [44]–[46]. Second, there may be an accelerated maturation rate of CD4^+^CD8^+^ double positive cells in the thymus, which is supported by the significant increase of thymic CD4^+^ T cells percentage observed in LPi mice. Third, apoptotic events may play a role in the decrease of double positive cells [24], [27], [42]. However, additional data are necessary to support each hypothesis. Also, we cannot rule out the possibility that alterations in other thymic cell populations could account for the loss of cellularity observed in LPi mice.

Interestingly, the significant increase of CD4^+^ T cell percentage in the thymus may also be due to altered migratory capabilities of these cells, which causes their accumulation and avoids migration to secondary organs. Based on the atrophy of the thymus in protein malnourished-infected mice, which results in the loss of weight and cellularity, it is suggested that components of the extracellular matrix and adhesion molecules may also be altered, compromising the migratory abilities necessary for adequate intra-thymic maturation, extra-thymic activation, lymphocyte proliferation, and ultimately, an appropriate immune response [47]–[49].

Our results show that protein malnutrition also dysregulated cytokine mRNA expression in the thymus of LPi animals. The defective regulation of thymic cytokine levels is intriguing. IL-4, IL-10 and IL-12a expression in the thymocytes of LPi mice were almost undetectable due to an interaction of low protein diet and infection, whereas TGF-β was increased due only to the effect of low protein diet. TGF-β, IL-10 and IL-4 are involved in the control of proliferation during the selection of thymocyte clones [50]; therefore, the altered expression of these cytokines may have deleterious consequences in this control and ultimately in thymic cell subpopulations. In activated thymic cells, expression of IL-12 has an enhancing effect on IL-10, IFN-g and TGF-β levels [50]. Accordingly, our results indicate that diminished IL-12 levels lead to diminished IL-10 and IFN-g levels in LPi mice. However, these animals showed increased levels of TGF-β, suggesting that this cytokine could be induced by other thymic factors such as the epidermal growth factor (EGF) [50]. The increase of TGF-β in LPi animals could account for a protecting role against apoptotic T cell events in thymus under nutritional and infection stresses.

Moreover, it is remarkable that IL-12 levels in the thymus may be regulated by infection. We observed that well-nourished animals presented high IL-12a mRNA expression in response to *L. infantum* infection, but LPi animals displayed diminished levels in response to the parasite. Remarkably, protein levels of secreted IL-12 were increased in CPi thymocytes, reinforcing the idea that the expression of this cytokine may be regulated by *Leishmania* infection. This response to parasite infection was abrogated in the thymocytes of LPi animals. As observed for other infections, IL-12 expression in the thymus appears to play a critical role in the recruitment of activated peripheral T cells for thymus re-entry when the total thymocyte numbers are reduced [51], [52]. In our model, because of the decreased level of IL-12 in the LPi mice, T cell re-entry to the thymus may be abrogated.

In addition, as IL-10 is involved in T cell proliferation in the thymus, the decrease in mRNA and protein levels of this cytokine in LPi mice may lead to a defective T cell proliferation in these animals. Dietary protein has been shown to be critical in sustaining T cell proliferation and memory because of the metabolic demand for an amino acid supply to support protein synthesis [53]. Therefore, our data may suggest that the decrease of thymic T cells could be a consequence of impaired proliferative capabilities in mice suffering protein malnutrition. Although preliminary, our data represent the first evidences on the thymus role during *Leishmania* infection in a protein malnourished murine model. Further studies will clarify the role of this lymphoid organ in our model.

Our histopathological findings are consistent with a severe structural disorganization of the splenic tissue in LPi animals. Separately, the low protein diet or the *L. infantum* infection increases the risk of having a moderate to highly disorganized tissue. However, the combination of these two factors remarkably increases such risk. In fact, that risk reach an *OR* = 105.0, indicating a synergistic deleterious effect of those conditions. Disruption of splenic architecture by protein malnutrition could turn more vulnerable this tissue to parasite infection. In turn, *L. infantum* infection promotes the disintegration of the splenic white pulp and the decrease or absence or germinal centers, among others [54]–[56]. Therefore, disorganization of the splenic architecture, as a combined result of protein restriction and *L. infantum* infection, must have deleterious consequences on the immune responses to the parasite. In fact, we observed a higher parasite load in LPi animals when compared to the CPi mice. In our model of protein malnutrition, even within a short course of infection (two weeks), we observed that the parasite load in liver and spleen reflects that what should be observed at later stages of infection in a typical BALB/c model [55], [57], indicating that protein malnutrition accelerates the events observed during a normal course of infection. In addition, protein malnutrition may have other deleterious impact on the parasite control in the liver as any granuloma formation could be observed in our model.

As reported by others, protein malnutrition induces an increase in CD4^+^ and CD8^+^ T cells in spleen; however, such populations present a defective proliferation [58]. In agreement with those results, we observed that LP and LPi mice exhibit an increase of splenic CD4^+^ and CD8^+^ T cells percentage, which should be beneficial for parasite control, as such T cells are critical for the primary *Leishmania* infection resolution [59]–[65]. Nevertheless, the functionality of these populations in LPi mice must be compromised, as a higher parasite load was observed in these animals in comparison to the control ones. In addition to the high splenic parasite load, we also observed dysregulation of cytokine mRNA expression induced by protein malnutrition in splenocytes of *L. infantum-*infected mice. We observed that protein malnutrition promotes a proinflammatory expression profile in the spleens of non-infected animals by increasing IL-12a and IFN-γ and decreasing TGF-β mRNA. By contrast, LPi mice exhibit a mixed response characterized by increasing IFN-γ and IL-10 mRNA levels and decreasing IL-12. Intriguingly, protein levels of IL-12, IFN-γ and IL-10 were undetectable in LPi mice, suggesting a defect on protein synthesis and/or exportation of these cytokines. In fact, it has been reported that nutrient starvation alters mRNA translation in murine models [66], compromising protein synthesis and therefore protein exportation.

Taken together, our results provide new evidences for immunological deficits caused by protein malnutrition in *L. infantum*-infected mice. Remarkably, we show that the thymus may play a critical role in the response to *L. infantum* infection when a protein malnutrition condition is pre-existent. We also show changes in the T cell populations from lymphoid organs, acceleration of the time course of infection, with pronounced histopathological damages and increased splenic parasite load, all of which may contribute for the impairment of the immune response against *L. infantum*.

## Supporting Information

S1 Table **Sequences of primers used for real time qPCR.**
(DOCX)
