# Supplementary material for: T-Cell Populations and Cytokine Expression Are Impaired in Thymus and Spleen of Protein Malnourished BALB/c Mice Infected with Leishmania infantum
Source: PLoS One. 2014 Dec 23;9(12):e114584. doi: 10.1371/journal.pone.0114584 (PMC4275170; doi:10.1371/journal.pone.0114584)
Supplement: S1 Table — Sequences of primers used for real time qPCR. (DOCX) [file pone.0114584.s001.docx]

**Table S1**. Sequences of primers used for real time qPCR

| **Accession**  **Number** | **Target** | **Forward Primer** | **Reverse Primer** | **Product size (bp)** | **Slope** | **Efficiency**  **(E) (%)** | **Correlation coefficient (R^2^)** |
| --- | --- | --- | --- | --- | --- | --- | --- |
| NM_011577.1 | ***TGF-β*** | ATTCCTGGCGTTACCTTGG | CCTGTATTCCGTCTCCTTGG | 117 | 3.33 | 99 | 0.95 |
| NM_008337.3 | ***IFNϒ*** | AGGACACAACAAGATGGA | TTAGTGAGAGTGAACATTACAG | 141 | 3.34 | 99 | 0.95 |
| NM_001278601.1 | ***TNF-α*** | CTTCCTACCTTCAGACCTT | GCCTTCCAAATAAATACATTCAT | 153 | 3.27 | 102 | 0.96 |
| NM_021283.2 | ***IL-4*** | AACGAAGAACACCACAGA | TCCATGAGAACACTAGAGTT | 117 | 3.27 | 102 | 0.96 |
| NM_010548.2 | ***IL-10*** | ACAACATACTGCTAACCGACTCCT | GGCAACCCAAGTAACCCTTAAAGT | 57 | 3.31 | 100 | 0.99 |
| NM_001159424.1 | ***IL-12a*** | CTCTCATATTCACTATACAAGTTG | GCTCTTCTGCTAACACAT | 94 | 3.31 | 100 | 0.98 |
| NM_001289726.1 | ***Gapdh*** | GCCTTCCGTGTTCCTACC | CTTCACCACCTTCTTGATGTC | 96 | 3.31 | 100 | 0.97 |
| NM_025567.2 | ***Cyc1*** | GGTGTCATTGCGAGAAGG | GGTGCCATCATCATACTCC | 106 | 3.32 | 100 | 0.99 |
| NM_016774.3 | ***ATPβ5*** | TGAGTGTTGAGCAGGAGATTC | TTGGCGACATTGTTGATTAGC | 148 | 3.39 | 97 | 0.99 |
| AF285161.1 | ***UBC*** | CTGTGAGAGCCGTGGATATTGG | GCACTTCCGTCTTTCAGCAAA | 84 | 3.47 | 94 | 0.98 |
| AF169140.1 | ***MiniC**** | TGTTGGTTGTGTGACTTTA | CATCCCACCAGACTAATC | 74 | 3.26 | 102 | 0.98 |

******* Taqman probe: FAM 5’-CTCCACCAGGCTCCAACC-3’-MGB
